# Comparative Mitogenomics of Jumping Spiders with First Complete Mitochondrial Genomes of Euophryini (Araneae: Salticidae)

**DOI:** 10.3390/insects14060517

**Published:** 2023-06-02

**Authors:** Wenqiang Zhang, Long Lin, Yuhui Ding, Feng Zhang, Junxia Zhang

**Affiliations:** Key Laboratory of Zoological Systematics and Application of Hebei Province, Institute of Life Science and Green Development, College of Life Sciences, Hebei University, Baoding 071002, China; zhangwq97@foxmail.com (W.Z.); linnlong0716@163.com (L.L.); yuhuiding4@gmail.com (Y.D.)

**Keywords:** mitogenome, gene arrangement, *Corythalia*, *Parabathippus*, *Colopsus*

## Abstract

**Simple Summary:**

Salticidae, a highly diverse lineage of spiders with 671 genera and 6497 species reported worldwide, are ideal model organisms for ecological, behavioral and evolutionary studies. Euophryini, one of the largest tribes of jumping spiders, lacks information on the features of mitochondrial genomes based on completely annotated data. Here, we sequenced and assembled the mitochondrial genomes of two euophryine species, *Corythalia opima* (G. W. Peckham & E. G. Peckham, 1885) and *Parabathippus shelfordi* (G. W. Peckham & E. G. Peckham, 1907). By comparing all available well-characterized mitogenomes of 13 salticid species, the common features and differences in the mitochondrial genomes of jumping spiders were explored. The implications of mitogenomic features for the evolution and taxonomy of jumping spiders were investigated.

**Abstract:**

Salticidae is the most species-rich family of spiders with diverse morphology, ecology and behavior. However, the characteristics of the mitogenomes within this group are poorly understood with relatively few well-characterized complete mitochondrial genomes. In this study, we provide completely annotated mitogenomes for *Corythalia opima* and *Parabathippus shelfordi*, which represent the first complete mitogenomes of the tribe Euophryini of Salticidae. The features and characteristics of the mitochondrial genomes are elucidated for Salticidae by thoroughly comparing the known well-characterized mitogenomes. The gene rearrangement between *trnL2* and *trnN* was found in two jumping spider species, *Corythalia opima* and *Heliophanus lineiventris* Simon, 1868. Additionally, the rearrangement of *nad1* to between *trnE* and *trnF* found in *Asemonea sichuanensis* Song & Chai, 1992 is the first protein-coding gene rearrangement in Salticidae, which may have an important phylogenetic implication for the family. Tandem repeats of various copy numbers and lengths were discovered in three jumping spider species. The codon usage analyses showed that the evolution of codon usage bias in salticid mitogenomes was affected by both selection and mutational pressure, but selection may have played a more important role. The phylogenetic analyses provided insight into the taxonomy of *Colopsus longipalpis* (Żabka, 1985). The data presented in this study will improve our understanding of the evolution of mitochondrial genomes within Salticidae.

## 1. Introduction

Mitochondrial genomes have been widely applied in studies, for example on the molecular evolution and the phylogeny of various animal lineages, because of their highly conserved structure, low recombination frequency and rapid evolutionary rate [1,2,3]. The mitochondrial genome of Metazoa is a circular, double-stranded molecule that typically consists of 13 protein-coding genes (PCG), 22 transfer RNA genes (tRNA), 2 ribosomal genes (rRNA) and a large non-coding control region (CR, also known as an AT-rich region) [4]. Certain features of mitogenomes, e.g., gene order pattern, nonsynonymous substitution vs. synonymous substitution rates of PCGs (Ka/Ks value), codon usage pattern, etc., in addition to the mitochondrial sequences, have proved to be informative in uncovering the phylogenetic relationships and understanding the evolutionary pressures in different organisms, such as nematodes [5], insects [6,7] and mites [8]. In addition, mitochondrial genes have shown great potential in DNA barcoding [9], detecting prey content [10], insecticide resistance [11] and metabolic differences [12]. Two notable features have been noticed in arachnid mitochondrial genomes: severely truncated tRNAs [13,14] and frequent gene rearrangements [15,16]. A total of 12 gene rearrangement patterns have been discovered in spider mitogenomes, all of which involved only tRNA and/or CR, with no PCG or rRNA rearrangements detected [17]. Li et al. [17] recently presented the largest mitochondrial phylogenomic study on spiders with a taxon sampling of 78 species and 29 families, and showed that both mitogenomic sequences and rearrangements were efficient for providing phylogenetic signals for spider phylogeny and characterizing trait diversification in spider evolution.

Salticidae, a highly diverse lineage of spiders with 671 genera and 6497 species reported worldwide [18], are well known for their large headlight-like eyes and excellent vision, and are ideal model organisms for ecological, behavioral and evolutionary studies [19]. However, the mitogenomes of jumping spiders have been poorly investigated. Currently, only 18 mitochondrial genomes of jumping spiders are available in the NCBI repository, of which 9 are complete and fully annotated, and the other 9 are unverified. So far, no complete mitogenome has been reported for the Euophryini, the most species-rich tribe of Salticidae with about 120 genera and over 1000 species known worldwide [19]. Previous studies on the mitogenomes of jumping spiders were usually scattered reports of mitogenomes for one or two species, such as *Phintella cavaleriei* (Schenkel, 1963) [20], *Epeus alboguttatus* (Thorell, 1887) [21] and *Habronattus oregonensis* (G. W. Peckham & E. G. Peckham, 1888) [22]. Therefore, the common features and differences of jumping spider mitogenomes are largely unexplored.

The limited mitochondrial genomic data of the Salticidae have prevented extensive comparative studies of mitochondrial genome sequences, gene arrangement and molecular evolution among various lineages within jumping spiders. In this study, we newly sequenced and annotated the mitochondrial genomes of *Corythalia opima* and *Parabathippus shelfordi*, and provided for the first time the complete mitogenome data for the tribe Euophryini. The common features, such as nucleotide composition, gene order, codon usage, nucleotide diversity, pairwise distance, etc., were analyzed and compared among the well-characterized jumping spider mitogenomes to lay out the foundation for future, more comprehensive comparative mitogenomic studies with an extended sampling of jumping spiders. The phylogeny was inferred to show the potential of mitogenomic sequences for providing valuable insight into the taxonomy of Salticidae.

## 2. Materials and Methods

### 2.1. Taxon Sampling

The complete mitochondrial genomes were obtained for two species of the tribe Euophryini. In addition, 11 of the 18 available salticid mitochondrial genomes that are complete or only lack part of control region, as well as 8 mitochondrial genomes of outgroups (other spider families), were downloaded from the Genbank (see Table 1 for detailed information). Specimens of the two euophryine species, *Corythalia opima* and *Parabathippus shelfordi*, were collected from Estación de Biología Los Tuxtlas, Veracruz, Mexico (18.585° N, 95.075° W; 13–18 July 2014) and Old Upper Thomson Road, Singapore (1.379° N, 103.816° E; 8 June 2019), respectively. All specimens were preserved in 95% ethanol and stored at −20 °C. The voucher specimens were deposited at the Museum of Hebei University, Baoding, China (MHBU) with the codes JXZ418 (*Corythalia opima*) and JXZ417 (*Parabathippus shelfordi*).

### 2.2. DNA Extraction, Library Preparation and Sequencing

Genomic DNA was extracted from the cephalothorax and legs using QIAGEN DNeasy Blood & Tissue Kit (Hilden, Germany), and RNA was removed with QIAGEN RNase A (Hilden, Germany). The quantity of DNA was checked using a Qubit^TM^ fluorometer (Singapore). The genomic DNA was sent to Novogene Co. Ltd. (Tianjin, China) for library preparation using the Truseq Nano DNA HT sample preparation kit (Illumina, San Diego, CA, USA), and then sequenced on the Illumina NovaSeq 6000 platform with 150 bp paired-end reads and insert size around 350 bp. About 5 Gb of raw data was obtained for each species to assemble the mitochondrial genome.

### 2.3. Mitochondrial Genome Assembly, Annotation and Sequence Analysis

After performing the quality control of raw reads to remove the adapters and low-quality reads (with ≤10% unidentified nucleotides, or with >50% bases having Phred quality <5, or with >10 nt aligned to the adapter, or read 1 and read 2 of the paired-end reads being completely identical), we proceeded with mitogenome assembly with the cleaned reads using MitoZ v3.4 [32,33] with the SPAdes assembler [34] and multi-kmer strategy (clade = Arthropoda; genetic_code = 5). The assembled complete mitogenomes were annotated with the online tool MITOchondrial genome annotation Server (MITOS; http://mitos.bioinf.uni-leipzig.de/index.py/ 10 February 2023) [35], and some tRNA loci were further annotated by ARWEN [36] and manual inspection. The secondary structures of tRNAs were predicted by MITOS and ARWEN, as well as manual inspection after determining the ends of adjacent protein-coding genes. The open reading frames (ORFs) of PCGs were examined in Geneious Prime^®^ 2023.0.1. The fully annotated circular maps of the two newly sequenced euophryine mitogenomes were then visualized in CHLOROPLO [37]. The above annotation procedures were also conducted on the two mitochondrial genomes, *Dendryphantes* sp. (MW832855) and *Heliophanus lineiventris* (MW832849), which were downloaded as “unverified” from the Genbank. In addition, the mitogenome of *Carrhotus xanthogramma* (Latreille, 1819) (KP402247) was re-annotated since Li et al. [17] suggested a different gene order from that of NCBI and the original publication [29]. The annotations for the other downloaded mitogenomes available in the Genbank were used in this study.

The software PhyloSuite v1.2.3 [38] and CodonW v1.4.4 (http://codonw.sourceforge.net/ 27 February 2023) were used to analyze the base composition, AT content, GC content, length, start codons, AT skewness, GC skewness, relative synonymous codon usage (RSCU) and effective number of codons (ENC) of the mitochondrial genomes. The nucleotide skewness values were calculated using the following formula: AT skew = [A − T]/ [A + T], GC skew = [G − C]/ [G + C] [39]. The 13 PCGs were aligned using MACSE v2 [40], and the aligned sequences were used to calculate the nonsynonymous substitution rates (Ka), synonymous substitution rates (Ks) and Ka/Ks of each PCG with the software KaKs_Calculator v3.0 with Model Averaging (MA) method [41]. The nucleotide diversity (Pi) of PCGs was calculated by a sliding window analysis using DnaSP v5.10 [42] with a window length of 100 bp and a step size of 25 bp. The pairwise genetic distances of each PCG were calculated by MEGA v11 [43] with the maximum composite likelihood method. The program Tandem Repeat Finder [44] (http://tandem.bu.edu/trf/trf.html 25 February 2023) was used to predict tandem repeats in the control region. The software IBM SPSS Statistics v26.0 was used to test the correlations between the effective codon (ENC) and average GC content in the third positions of PCG codons (GC3), and the average GC content in the first and second positions of PCG codons (GC12) and GC3, respectively.

### 2.4. Phylogenetic Analyses

The mitochondrial genome sequences of 21 spider species were included in the phylogenetic analyses, of which 13 were from the ingroup jumping spider species, and 8 were from the outgroup families (1 each of Cheiracanthiidae, Ctenidae, Lycosidae, Oxyopidae, Philodromidae, Pisauridae, Selenopidae and Trochanteriidae; Table 1). Both the nucleotide and amino acid sequences of the 13 PCGs were used for phylogenetic reconstruction.

The 13 PCG sequences were aligned, respectively, using MAFFT v7.505 [45] with the L-INS-i strategy, and the gaps and misaligned sites were trimmed in trimAl v1.2rev57 [46] with the “automated1” mode. The trimmed alignments were concatenated in PhyloSuite v1.2.3, and the ModelFinder v2.2.0 [47] was used to select the best partition and model with Bayesian information criterion (BlC). The maximum likelihood (ML) analyses were performed in IQ-TREE v2.2.0 [48] using the optimized model and partition scheme, and an ultrafast bootstrap [49] analysis with 1000 replicates was conducted to assess the node support. The Bayesian inference (BI) was completed using MrBayes v3.2.7 [50] with two independent runs (500 million generations and four chains in each run, sampling every 1000 generations). The resulting log files were imported into Tracer v1.7.2 [51] to check the convergence, and the trees sampled during the first 20% of generations were discarded as burn-in. The resulting trees were visualized via Figtree v1.4.4 (http://tree.bio.ed.ac.uk/software/figtree/ 20 March 2023).

## 3. Results

### 3.1. General Features of Mitogenomes of Euophryini

**Figure 1:**
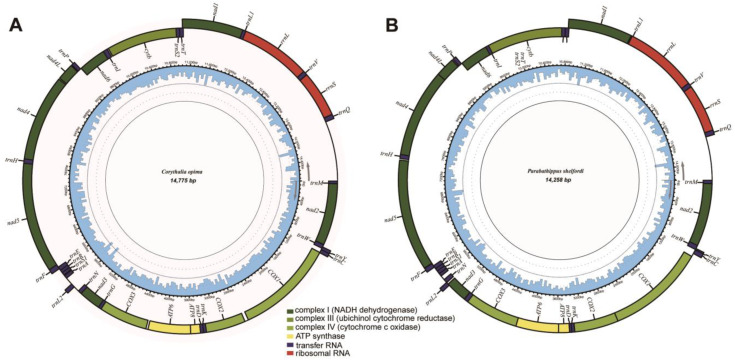
Circular map of the complete mitochondrial genome of (**A**) *Corythalia opima* and (**B**) *Parabathippus shelfordi*, with the majority (J) and minority (N) strands shown inside and outside of the circular map, respectively. The inner circle blue bar indicates the GC content of the locus.

**Figure 2:**
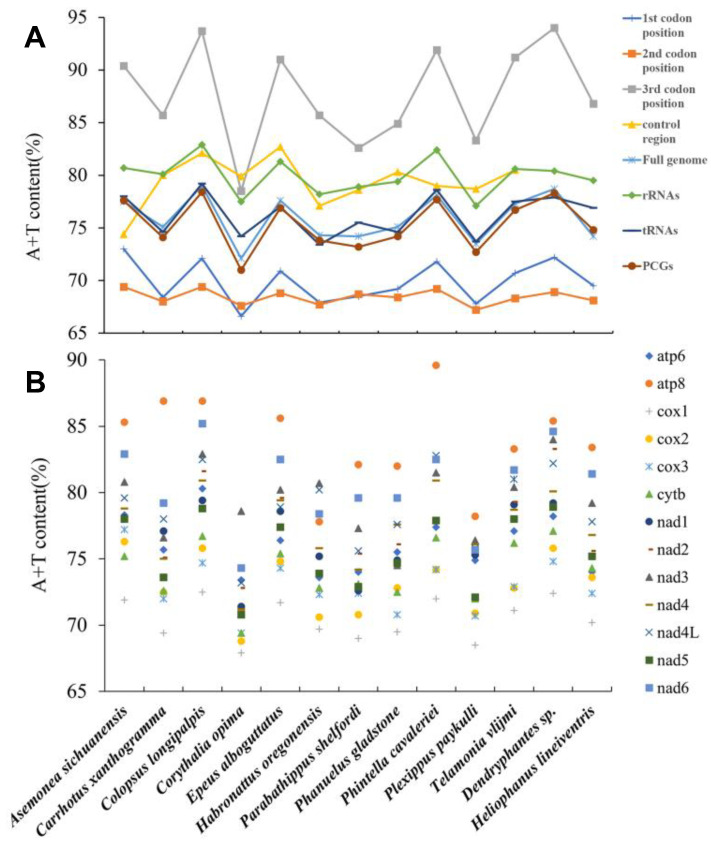
The A + T contents of (**A**) transfer RNAs (tRNAs), ribosomal RNAs (rRNAs), the whole, first, second, third positions of protein-coding genes (PCGs), control region and full mitogenome; and (**B**) the 13 PCGs in all jumping spider species (the control regions of *Dendryphantes* sp. and *Heliophanus lineiventris* are not complete, so their A + T contents are not provided).

This study presents the first report on the complete characterization of mitogenomes for the jumping spider tribe Euophryini. The assembled and annotated complete mitochondrial genomes of the two euophryine species were uploaded to Genbank with the accession numbers OQ281589 and OQ429315 (Table 1). The mitogenomes of *Corythalia opima* and *Parabathippus shelfordi* are 14,775 bp and 14,258 bp in length, respectively, which are close to the known mitogenome length of jumping spiders (14,316 bp to 15,419 bp; Supplementary Table S1). Consistent with other jumping spiders, the mitogenomes of Euophryini are double-stranded circular, comprising 22 tRNAs, 13 PCGs, 2 rRNAs and a non-coding control region (also known as an AT-rich region), of which 9 PCGs, 13 tRNAs and the control region are in the majority strand (J-strand), and 4 PCGs, 9 tRNAs and 2 rRNAs are in the minority strand (N-strand) (Figure 1). The base composition of the full mitogenome is A = 29%, T = 43.1%, C = 8.4%, G = 19.4% for *Corythalia opima*, and A = 32.9%, T = 41.3%, C = 8.6%, G = 17.2% for *Parabathippus shelfordi*, and both species have high A + T content (72.1% and 74.2%, respectively). All jumping spider species with complete mitogenomes have the highest A + T content in the third codon positions, except *Corythalia opima*, which has the highest A + T content in the control region (Figure 2A). Similar to other jumping spiders, the two euophryine species show typical negative AT skews (−0.196 and −0.113, respectively) and positive GC skews (0.394 and 0.334, respectively) in the full mitogenome.

### 3.2. Protein-Coding Genes

The total length of 13 PCGs of the jumping spider mitogenomes range from 10,611 bp (*Heliophanus lineiventris*) to 10,812 bp (*Parabathippus shelfordi*), with four PCGs (*nad1*, *nad4*, *nad4L* and *nad5*) in the N-strand and the remaining nine PCGs (*atp6*, *atp8*, *cox1*, *cox2*, *cox3*, *cytb*, *nad2*, *nad3* and *nad6*) in the J-strand (Supplementary Tables S1 and S2). Within jumping spiders, the longest PCG is *nad5* (1575–1644 bp), and the shortest is *atp8* (144–168 bp). Of the 13 PCGs, *nad4L* shows the highest variation in length among jumping spiders (225–318 bp). The two PCGs, *cox3* and *atp6*, are more conservative in length, with most species being around 786 bp (*cox3*) and 666 ± 3 bp (*atp6*), respectively. Most of the start codons in the mitogenomes of Salticidae are ATN or TTN, and the unconventional start codons (i.e., CTG, GTA, CGA and AGA) only occur in some *cox1*; the stop codons are usually TAA or TAG, but are truncated to T in some, i.e., *atp6*, *cytb*, *cox2*, *cox3*, *nad2*, *nad4*, *nad4L*, *nad5* and *nad6*, or to TA in the *nad6* of *Plexippus paykulli* (Audouin, 1826) (Supplementary Table S1).

The PCGs in jumping spider mitogenomes show a typical AT preference with the A + T content of all PCGs ranging from 71.0% to 78.4% (Supplementary Table S2), and the third codon positions having much higher A + T content (78.5–94.0%) than the first and second codon positions (66.6–73.0%; Figure 2A). Among the 13 PCGs, *atp8* has higher A + T content than other PCGs in all jumping spider species except *Corythalia opima* and *Habronattus oregonensis*, in which *nad3* has the highest A + T content, and *cox1* has the lowest A + T content (Figure 2B; Supplementary Table S3). The nucleotide skewness analysis shows that the PCGs of all jumping spider species prefer T and G over A and C with AT skewness from −0.166 to −0.121 and GC skewness from 0.036 to 0.108 (Supplementary Table S2).

**Figure 3:**
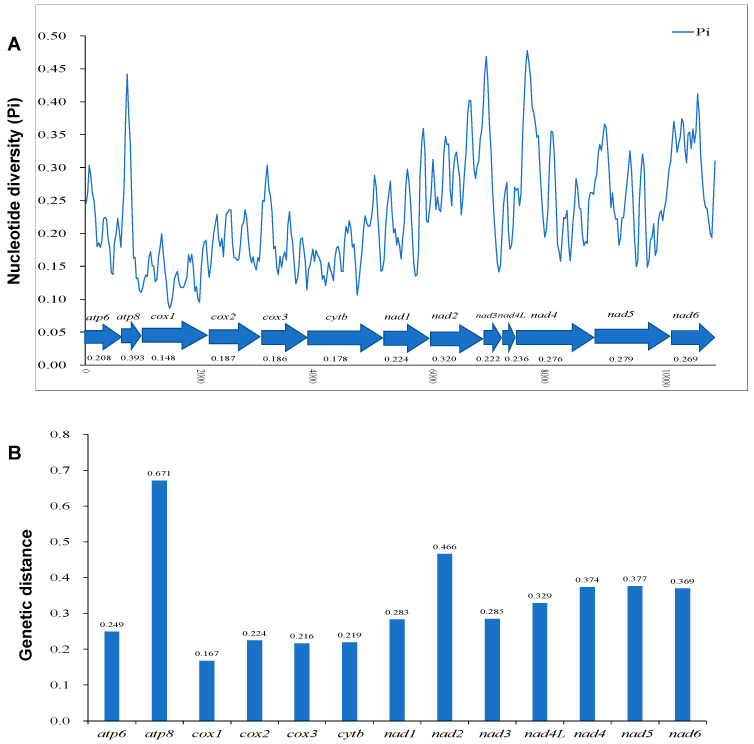
(**A**) The nucleotide diversity (Pi) of 13 protein-coding genes (PCGs) in Salticidae mitogenomes determined via sliding window analysis (sliding window: 100 bp; step size: 25 bp); the Pi value of each gene is shown under the gene name. (**B**) Pairwise genetic distances of 13 PCGs among jumping spider species. The average value for each PCG is shown on the graph.

The sliding window analysis shows that the nucleotide diversity (Pi) of the 13 PCGs in Salticidae is highly variable, with the highest Pi for *atp8* (0.393) followed by *nad2* (0.320) and *nad5* (0.279), and the lowest Pi for *cox1* (0.148) (Figure 3A). The analysis of pairwise genetic distances among the jumping spider species shows that among the 13 PCGs, *atp8* (0.671) and *nad2* (0.466) have evolved relatively quickly, while *cox1* (0.167) relatively slowly (Figure 3B).

**Figure 4:**
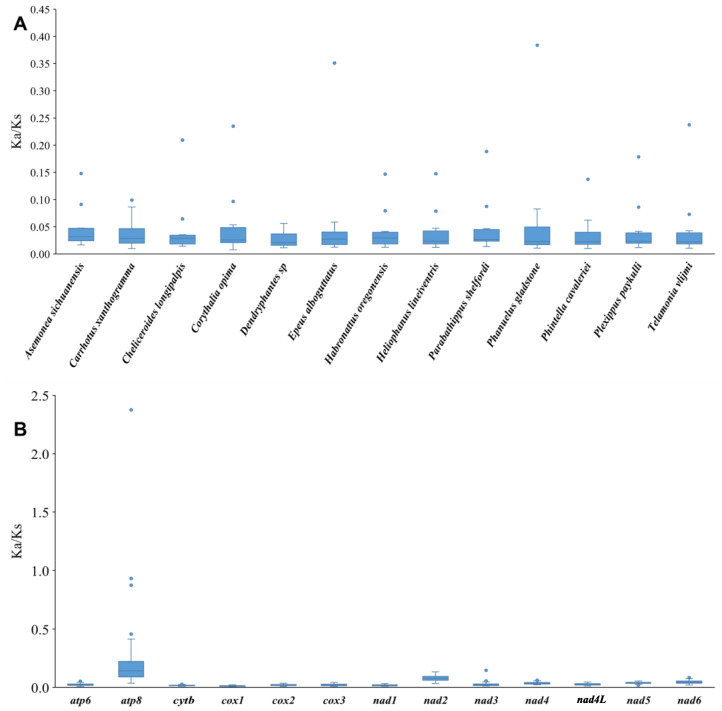
Boxplots of (**A**) Ka/Ks for the 13 mitochondrial protein-coding genes (PCGs) and (**B**) Ka/Ks of the 13 PCGs across the 13 salticid mitogenomes examined.

To estimate the evolutionary rate, the Ka/Ks values were calculated for the 13 PCGs of Salticidae. The results show that *atp8* has the highest mean Ka/Ks value, which indicates it may have evolved more rapidly than the other PCGs in Salticidae, whereas *cox1* has the lowest mean value of Ka/Ks, implying a slower rate of evolution (Figure 4A). Some Ka/Ks values of *atp8* are greater than 1 in *Epeus alboguttatus* and *Phanuelus gladstone* Caleb & Mathai, 2015, and are close to 1 in *Dendryphantes* sp. and *Colopsus longipalpis*. Comparing the mean Ka/Ks values of PCGs among salticid species, the results show that the mean Ka/Ks values of all species are below 1 and similar to each other (Figure 4B).

The codon usage analyses showed that 62 available codons are used in the 13 PCGs of jumping spiders, although some species have 60 or 61 available codons. The relative synonymous codon usage and the number of each amino acid for each salticid species are shown in Supplementary Figure S1. The preferred codon in Salticidae species is UUA, and the most frequently used amino acids are Leu2, Ile, Met and Phe. In the two euophryine species, *Corythalia opima* prefers the codons UUA, GCU, CCU and UCU, while *Parabathippus shelfordi* prefers the codons UUA, CCU, GCU and UCU.

**Figure 5:**
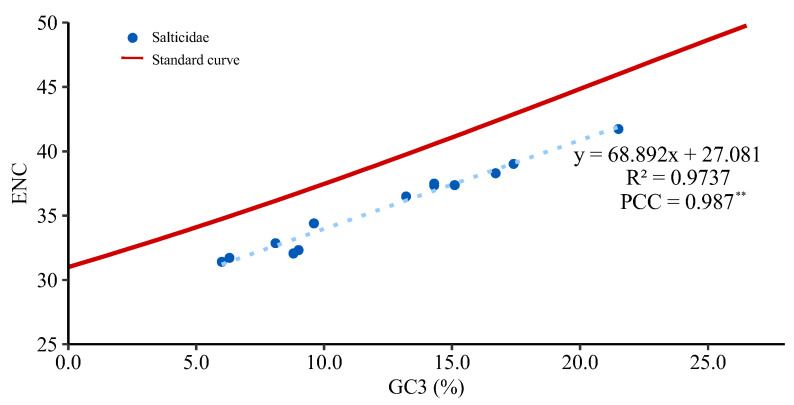
The plot of effective codon (ENC) and average GC content in the third positions of PCG codons (GC3) in the mitogenomes of 13 salticid species. The standard curve describes the relationship between the ENC and GC3 without selection pressure (Pearson correlation coefficient = PCC; ** indicates statistically significant at *p* < 0.01).

**Figure 6:**
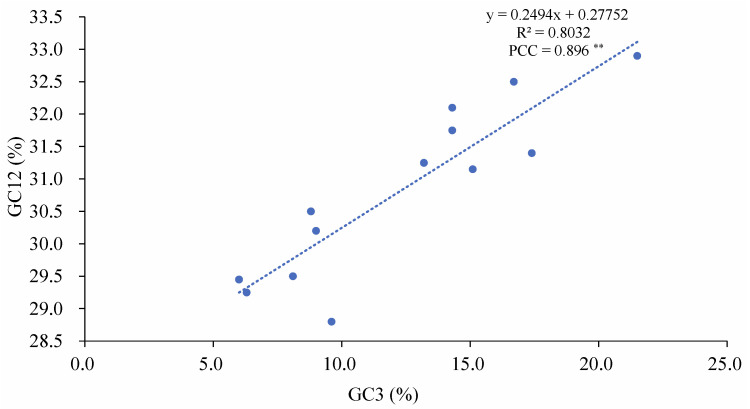
Neutrality plot analysis of the average GC content in the first and second positions (GC12) and that in the third positions (GC3) of PCG codons in the mitogenomes of 13 salticid species (Pearson correlation coefficient = PCC; ** indicates statistically significant at *p* < 0.01).

The number of effective codons (ENCs) ranges from 31.41 to 41.73 in the 13 PCGs of Salticidae (Supplementary Table S4), indicating a variation in codon usage bias among jumping spiders. Some species, such as *Colopsus longipalpis* (31.72) and *Dendryphantes* sp. (31.41), have an ENC ≤ 35 and show significant codon usage bias, while others, such as the two euophryine species (*Corythalia opima*, 41.73; *Parabathippus shelfordi*, 39.01) have an ENC > 35 and show no significant codon usage bias. The ENC-GC3 plot (Figure 5) shows a positive correlation between ENC and GC3 (Pearson correlation coefficient = 0.987, *p* < 0.01) with the distribution of jumping spider species below the standard curve. The GC12 values range from 0.288 to 0.329 and GC3 values from 0.06 to 0.215 in the jumping spider species (Supplementary Table S4), and the neutral plot (Figure 6) shows a positive correlation between GC12 and GC3 (Pearson correlation coefficient = 0.896, *p* < 0.01).

### 3.3. Transfer and Ribosomal RNA Genes

The mitochondrial genomes of jumping spiders contain 22 tRNA genes, 1 for each amino acid, with an additional isoform for each of serine and leucine. Of the 22 tRNA genes, 13 are on the J-strand and the remaining nine are on the N-strand (Supplementary Table S1). The length of the 22 tRNAs in the jumping spiders ranges from 1144 bp to 1342 bp, and their AT content ranges from 73.4% to 79.2%. The tRNAs in jumping spiders do not show strong A/T bias with AT-skew values close to 0, but they clearly prefer G over C with GC-skew values ranging from 0.129 to 0.271 (Supplementary Table S2). The gene order in jumping spider mitogenomes is conserved, but a rearrangement between *trnL2* and *trnN* is found in *Corythalia opima* and *Heliophanus lineiventris*, and a rearrangement of *nad1* to between *trnE* and *trnF* is found in *Asemonea sichuanensis*.

The predicted secondary structures of the 22 tRNAs for the two Euophryini species are shown in Supplementary Figures S2 and S3, respectively. Some tRNAs fail to fold into the typical cloverleaf-shaped secondary structures, as observed in the mitochondrial genomes of many arachnids [13,22]. The *trnS1* of both Euophryini species lacks the dihydrouridine (DHU) arm, which is a common feature of most metazoans [3]. There are mismatched base pairs in the secondary structures of tRNAs, including U-U, U-C, A-C, T-G, A-A, A-G, G-G, A-C and C-C, with the mismatches occurring mainly in the amino acid (AA) arms and the TΨC arms.

The mitogenomes of jumping spiders have two rRNAs, *rrnL* (between *trnL1* and *trnV*) and *rrnS* (between *trnV* and *trnQ*), ranging in length from 1419 bp to 1822 bp and with A + T content from 77.1% to 82.9%. In most jumping spiders, the rRNAs show positive AT skews (0.01–0.148) and negative GC skews (−0.21–−0.003), but in *Dendryphantes* sp. and *Epeus alboguttatus* the rRNAs show positive GC skews (0.022 and 0.006, respectively; Supplementary Table S2).

### 3.4. Control Region

**Figure 7:**
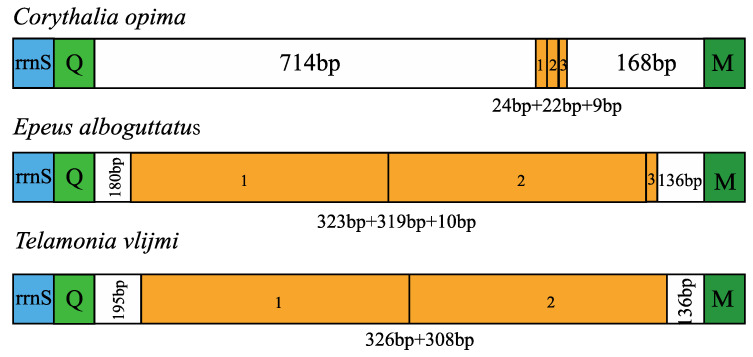
Repeat units of the control region of the mitochondrial genome from *Corythalia opima*, *Epeus alboguttatus* and *Telamonia vlijmi*. rrnS and tRNAs are indicated by the blue and green square bars, respectively.

The control region (CR), also known as the AT-rich region with A + T content ranging from 74.4% to 82.7%, is located between *trnQ* and *trnM* in the jumping spider mitogenomes, and has typical negative AT skew and positive GC skew, indicating a preference for T and G. The control region of *Asemonea sichuanensis* (1793 bp) is significantly longer than that of other jumping spider species (657 bp to 968 bp) (Supplementary Table S2). The tandem repeats are found in the control regions of three jumping spider species with variation in the copy number and length (Figure 7): the CR of *Corythalia opima* has one tandem repeat of 24 bp with two partial sequences of 22 bp and 9 bp, *Epeus alboguttatus* has one tandem repeat of 323 bp with two partial sequences of 319 bp and 10 bp, and *Telamonia vlijmi* Prószyński, 1984, presents one tandem repeat of 326 bp with a partial sequence of 308 bp. No tandem repeats are observed in the other well-characterized mitogenomes of Salticidae.

### 3.5. Phylogenetic Analyses

**Figure 8:**
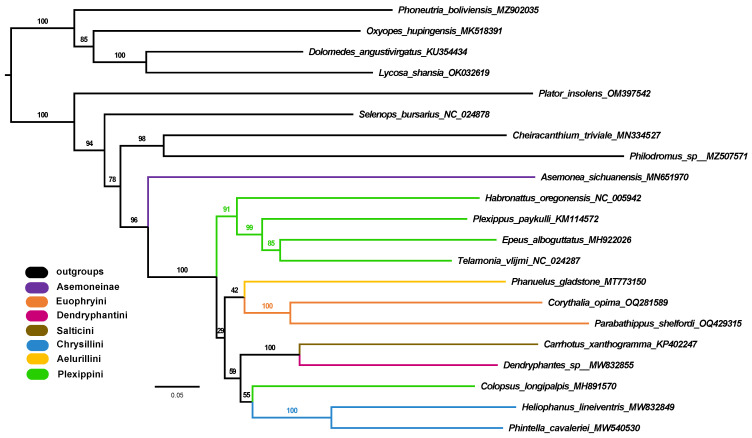
Phylogenetic tree from the ML analysis of the nucleotide dataset, with the numbers on the branches indicating bootstrap supports.

The results of phylogenetic analyses from the nucleotide and amino acid sequences of the 13 PCGs are shown in Figure 8 and Supplementary Figures S4–S6. The BI and ML analyses on the nucleotide dataset recovered the same topology, in which the monophyly of Salticidae (posterior probability, pp = 1; bootstrap, bs = 96%) and Salticinae (pp = 1; bs = 100%) are supported. Both analyses suggested the sister relationship of Salticidae with Cheiracanthiidae + Philodromidae, but this is not well supported (pp = 0.6; bs = 78%). Within Salticinae, the tribe Plexippini is rendered paraphyletic with *Colopsus longipalpis*.

## 4. Discussion

The size and content of mitochondrial genomes are conserved in Salticidae, including Euophryini, as shown in the other groups of spiders [17]. The nucleotide skewness analysis is commonly used to reveal the nucleotide composition dynamics of mitogenomes [39,52,53], which shows that the mitogenomes as a whole in jumping spiders are clearly AT-biased with much higher A + T content than G + C content, and often prefer T over A and G over C with negative AT skews and positive GC skews. This has been found repeatedly in other spiders, such as *Leucauge celebesiana* (Walckenaer, 1841) [54], *Argiope perforata* Schenkel, 1963 [55] and *Argiope ocula* Fox, 1938 [56]. The *apt8* has the highest A + T content among the mitochondrial PCGs in almost all salticid species examined except *Corythalia opima* and *Habronattus oregonensis*, but its significance still needs further investigation with extended sampling in a comparative mitogenomic study. 

**Figure 9:**
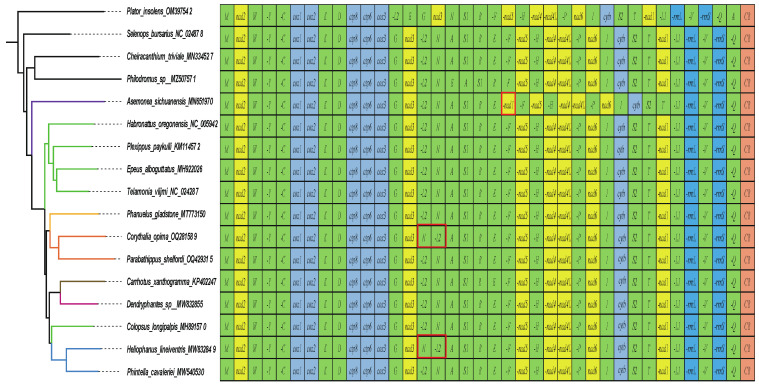
Genomic rearrangements of Salticidae. The left phylogenetic tree is from the ML analysis of the nucleotide dataset, and the red box lines represent the rearrangements of tRNAs and PCG within jumping spiders.

Genomic rearrangements, which have been considered useful markers for deep phylogenetic inference in some lineages, are relatively common in arachnids [55,56]. Two types of gene arrangements have been found in well-characterized jumping spider mitogenomes (Figure 9). One type of rearrangement occurs between the *trnL2* and *trnN* in *Corythalia opima* (Euophryini) and *Heliophanus lineiventris* (Chrysillini), which has also been discovered in other spider families such as Desidae (*Desis jiaxiangi* Lin, Li & Chen, 2020 [15]). This type of rearrangement can be classified as shuffling because the genes did not cross PCGs but moved from their original positions to the adjacent positions. As reported in previous studies on spider mitogenomic gene arrangement [57,58], the tRNA rearrangements in jumping spiders can be explained by the tandem duplication and random loss model (TDRL), which assumes that the rearrangement of mitochondrial genes occurs through the tandem duplication of specific genes, followed by the random loss of one copy of each gene [59,60]. This mechanism would change the position of the gene but not its orientation.

The other type represents the first PCG rearrangement of the mitochondrial genomes in spiders, which involves rearranging *nad1* to between *trnE* and *trnF*. So far, this rearrangement has only been observed in *Asemonea sichuanensis* (Asemoneinae), the only species of the basal lineages of jumping spiders with a well-characterized mitogenome. The basal lineages of jumping spiders comprise six subfamilies (Asemoneinae, Eupoinae, Hisponinae, Lyssomaneinae, Onomastinae and Spartaeinae) of Salticidae, all except the subfamily Salticinae that possesses the bulk species diversity of jumping spiders [19]. Resolving the relationships of these basal lineages is essential to clarify the jumping spider phylogeny. However, it is hard to determine if the rearrangement of *nad1* found in *Asemonea sichuanensis* is ancestral in jumping spiders and has phylogenetic implications due to the lack of knowledge on the mitogenomic features of other basal salticid lineages. 

tRNAs are key in translation, serving as adapter molecules between mRNA codons and amino acids [61]. All the examined jumping spider mitogenomes have 22 tRNAs. Although the loss of tRNA genes in animal mitochondrial genomes is rare, it has been reported in a variety of animal groups [62], including some arachnids such as scorpions [63], mites [64] and wolf spiders (*Pirata subpiraticus* (Bösenberg & Strand, 1906), lacking *trnG* [17]). Mismatches of base pairs in the AA arm are common in most tRNAs of spider mitogenomes, which might be a typical feature of spider mitochondrial tRNAs [17]. Previous studies have proposed that a fully paired AA arm in spiders may be formed by tRNA editing [13,22]. Lacking a DHU or TΨC arm in tRNAs is pretty common in spider mitogenomes [17], and truncated mitochondrial tRNAs may cause difficulty in the accurate annotation of certain tRNA genes [17]. However, the truncated secondary structure in certain tRNAs may not influence the translation process. For instance, a previous study has shown that in nematodes, mitochondrial tRNAs with extremely short structures, can still be recognized by synthesizing enzymes and can be aminoacylated [65].

The control region is an essential element involved in the replication and transcription initiation of the mitochondrial genome [66]. As the largest non-coding region, the length of the control region in jumping spiders varies, with that of *Asemonea sichuanensis* being almost twice as long as the other examined salticid species. However, this variation seems to not be completely due to the variation in the length and copy number of tandem repeats (TRs), since no TR is found in *Asemonea sichuanensis.* TRs have been found in other spider species, such as *Argiope amoena* L. Koch, 1878 [67], *Argiope bruennichi* [68] and *Trichonephila clavata* (L. Koch, 1878) [69]. However, among the 11 examined jumping spider mitogenomes with complete CR regions, TRs are present in only three species (*Corythalia opima*, *Epeus alboguttatus* and *Telamonia vlijmi*), indicating that the presence of TRs may not be a conserved feature, at least within Salticidae. The highly repetitive sequences (often A-T-rich) in the control region may inhibit DNA polymerases and lead to sequencing failures [70,71], which may account for the incomplete control regions in some registered salticid mitogenomes, e.g., *Heliophanus lineiventris* (MW832849) and *Dendryphantes* sp. (MW832855).

The PCGs of the jumping spider species share the same AT and GC skewness pattern as the full mitogenome. The mean Ka/Ks value for the PCGs is less than 1 in all the examined jumping spider species, indicating that the mitogenomes of the jumping spiders are generally subject to purifying selection, as documented in other organisms such as spider mites [72] and insects [73]. Among all the PCGs, the *atp8* and *nad2* exhibit relatively high nucleotide diversity (Pi), pairwise genetic distances and Ka/Ks values, with Ka/Ks values for *atp8* sometimes around or higher than 1 (Figure 4). This suggests these two genes may have evolved under a relatively relaxed purifying selection that may be related to an adaptation to new environments [74]. For instance, positive selection on genes such as *nad4*, *cytb* and *atp8* was believed to have acted to meet the enormous changes in energy demand and have played a pivotal role in the evolution of bat flight [75]. The *cox1* gene has the lowest Ka/Ks value and less amino acid variation, so it has been widely applied as a barcode marker in spiders and other animals [15]. Codon usage patterns are often influenced by selection and mutational pressures [76]. ENC and neutral maps are commonly used to detect the proportion of evolutionary pressure [77,78]. The ENC-GC3 plot (Figure 5) showed a distribution of the salticid PCGs below the standard curve, which indicates the codon usage bias in jumping spider mitogenomes is affected by natural selection [79]. The neutrality plot analysis showed a positive correlation between GC12 and GC3, indicating that mutational pressure also affects codon usage in the evolution of jumping spider mitogenomes. The slope of the regression line (0.249) suggested that natural selection (75.1%) plays a more important role in the evolution of amino acid composition in jumping spiders than mutational pressure (24.9%) [76]. 

Although the phylogenetic analyses in this study have few exemplar taxa due to the limited number of well-characterized mitogenomes, especially for jumping spiders, they show the potential of mitochondrial sequences for resolving the phylogenetic relationships of spiders and providing insight into the placement of certain problematic taxa. Both analyses on the nucleotide and amino acid datasets recovered the same relationships among the Salticidae and the outgroup families, which are largely congruent with recent transcriptome- or UCE (ultra-conserved element)-based phylogenomic studies [80,81,82,83]. Within the Dionycha clade, Salticidae was suggested to be a sister to the clade of Cheiracanthiidae + Philodromidae (Figure 8) through mitogenomic PCGs, but the phylogenomic study on Dionycha combining genomic, morphological and Sanger data indicated a sister relationship between Salticidae and Philodromidae [83]. This discrepancy is likely due to the limited taxon sampling in the mitogenomic dataset. Recent progress in molecular phylogenetic studies of jumping spiders, especially the application of the phylogenomic approach, has strongly promoted an understanding of the relationships of major salticid lineages [19,84]. However, only two (Asemoneinae and Salticinae) of the seven salticid subfamilies were included in the mitogenomic phylogenetic analyses of this study, in which the monophyly of the subfamily Salticinae was strongly supported. Thus, the performance of mitogenome sequences in reconstructing the phylogeny of salticids needs to be further tested with a more extended sampling of jumping spider mitogenomes. 

An interesting finding through the phylogenetic analyses with the mitogenomic PCGs is the placement of *Colopsus longipalpis*, which was not grouped with the other members of the tribe Plexippini in the resulting phylogeny (Figure 8). *Colopsus longipalpis* was originally classified in the genus *Cheliceroides* Żabka, 1985, and was designated as the type species [85]. Additionally, *Cheliceroides* was considered a member of the salticid tribe Hasariini [19]. However, Logunov synonymized *Cheliceroides* with *Colopsus* based on morphological characteristics, which resulted in the current placement of *Colopsus longipalpis* within the tribe Plexippini [86]. The mitogenomic phylogeny challenged Logunov’s taxonomic treatment and suggested that *Colopsus longipalpis* might not be a member of Plexippini, but further evidence is needed to clarify its taxonomic status and phylogenetic position.

## Figures and Tables

**Table 1:** Information of the representative taxa used in the phylogenetic analyses.

Family	Subfamily	Tribe	Species	GenBank Accession Number	Reference
Cheiracanthiidae			*Cheiracanthium triviale*	MN334527	[16]
Ctenidae			*Phoneutria boliviensis*	MZ902035	Unpublished
Lycosidae			*Lycosa shansia*	OK032619	[23]
Oxyopidae			*Oxyopes hupingensis*	MK518391	[24]
Philodromidae			*Philodromus* sp.	MZ507571	Unpublished
Pisauridae			*Dolomedes angustivirgatus*	KU354434	[25]
Salticidae	Asemoneinae		*Asemonea sichuanensis*	MN651970	Unpublished
	Salticinae	Aelurillini	*Phanuelus gladstone*	MT773150	Unpublished
		Chrysillini	*Heliophanus lineiventris*	MW832849	[17]
			*Phintella cavaleriei*	MW540530	[20]
		Dendryphantini	*Dendryphantes* sp.	MW832855	[17]
		Euophryini	*Corythalia opima*	OQ281589	This study
			*Parabathippus shelfordi*	OQ429315	This study
		Plexippini	*Colopsus longipalpis*	MH891570	[26]
			*Epeus alboguttatus*	MH922026	[21]
			*Habronattus oregonensis*	NC005942	[22]
			*Plexippus paykulli*	KM114572	[27]
			*Telamonia vlijmi*	NC024287	[28]
		Salticini	*Carrhotus xanthogramma*	KP402247	[17,29]
Selenopidae			*Selenops bursarius*	NC024878	[30]
Trochanteriidae			*Plator insolens*	OM397542	[31]

## Data Availability

The newly produced mitogenomes are publicly available in GenBank under the voucher numbers listed in Table 1.

## Supplementary Materials

The following supporting information can be downloaded at https://www.mdpi.com/article/10.3390/insects14060517/s1, Figure S1: The (A) relative synonymous codon usage (RSCU) and (B) number of amino acids in the 13 protein coding genes of salticid species. Codon families are labeled below the X-axis; Figure S2: Predicted secondary structures of tRNAs in the mitochondrial genome of Corythalia opima; Figure S3: Predicted secondary structures of tRNAs in the mitochondrial genome of Parabathippus shelfordi; Figure S4: Phylogenetic tree from the ML analysis of the amino acid dataset, with the numbers on the branches indicating bootstrap supports; Figure S5: Phylogenetic tree from the BI analysis of the amino acid dataset, with the numbers on the branches indicating posterior probabilities; Figure S6: Phylogenetic tree from the BI analysis of the nucleotide dataset, with the numbers on the branches indicating posterior probabilities; Table S1: Annotation of the mitochondrial genomes of the jumping spider species; Table S2: Nucleotide composition of mitogenomes of Salticidae; Table S3: The A + T contents of the 13 PCGs of Salticidae; Table S4: The GC12 content, GC3 content and ENC of PCGs of Salticidae.

## References

[1] Boore J.L. (1999). Animal mitochondrial genomes. Nucleic Acids Res..

[2] Boore J.L., Collins T.M., Stanton D., Daehler L.L., Brown W.M. (1995). Deducing the pattern of arthropod phylogeny from mitochondrial DNA rearrangements. Nature.

[3] Castellana S., Vicario S., Saccone C. (2011). Evolutionary patterns of the mitochondrial genome in Metazoa: Exploring the role of mutation and selection in mitochondrial protein-coding genes. Genome Biol. Evol..

[4] Wolstenholme D.R. (1992). Animal mitochondrial DNA: Structure and evolution. Int. Rev. Cytol..

[5] Kern E.M.A., Kim T., Park J.K. (2020). The mitochondrial genome in nematode phylogenetics. Front. Ecol. Evol..

[6] Song F., Li H., Jiang P., Zhou X., Liu J., Sun C., Vogler A.P., Cai W. (2016). Capturing the phylogeny of Holometabola with mitochondrial genome data and Bayesian site-heterogeneous mixture models. Genome Biol. Evol..

[7] Ge X.Y., Liu T., Kang Y., Liu H.Y., Yang Y.X. (2022). First complete mitochondrial genomes of Ototretinae (Coleoptera, Lampyridae) with evolutionary insights into the gene rearrangement. Genomics.

[8] Arribas P., Andújar C., Moraza M.L., Linard B., Emerson B.C., Vogler A.P. (2020). Mitochondrial metagenomics reveals the ancient origin and phylodiversity of soil mites and provides a phylogeny of the Acari. Mol. Biol. Evol..

[9] Lopardo L., Uhl G. (2014). Testing mitochondrial marker efficacy for DNA barcoding in spiders: A test case using the dwarf spider genus *Oedothorax* (Araneae: Linyphiidae: Erigoninae). Invertebr. Syst..

[10] Xu C.C.Y., Yen I.J., Bowman D., Turner C.R. (2015). Spider web DNA: A new spin on noninvasive genetics of predator and prey. PLoS ONE.

[11] Van Nieuwenhuyse P., Demaeght P., Dermauw W., Khalighi M., Stevens C.V., Vanholme B., Tirry L., Lümmen P., Van Leeuwen T. (2012). On the mode of action of bifenazate: New evidence for a mitochondrial target site. Pestic. Biochem. Physiol..

[12] Pichaud N., Ballard J.W.O., Tanguay R.M., Blier P.U. (2012). Naturally occurring mitochondrial DNA haplotypes exhibit metabolic differences: Insight into functional properties of mitochondria. Evolution.

[13] Masta S.E., Boore J.L. (2008). Parallel evolution of truncated transfer RNA genes in arachnid mitochondrial genomes. Mol. Biol. Evol..

[14] Pons J., Bover P., Bidegaray-Batista L., Arnedo M.A. (2019). Arm-less mitochondrial tRNAs conserved for over 30 millions of years in spiders. BMC Genom..

[15] Li F., Lv Y., Wen Z., Bian C., Zhang X., Guo S., Shi Q., Li D. (2021). The complete mitochondrial genome of the intertidal spider (*Desis jiaxiangi*) provides novel insights into the adaptive evolution of the mitogenome and the evolution of spiders. BMC Ecol. Evol..

[16] Tyagi K., Kumar V., Poddar N., Prasad P., Tyagi I., Kundu S., Chandra K. (2020). The gene arrangement and phylogeny using mitochondrial genomes in spiders (Arachnida: Araneae). Int. J. Biol. Macromol..

[17] Li M., Chen W.T., Zhang Q.L., Liu M., Xing C.W., Cao Y., Luo F.Z., Yuan M.L. (2022). Mitochondrial phylogenomics provides insights into the phylogeny and evolution of spiders (Arthropoda: Araneae). Zool. Res..

[18] Platnick N.I. (2023). The World Spider Catalog, Natural History Museum Bern, online at version 24.0. https://wsc.nmbe.ch.

[19] Maddison W.P. (2015). A phylogenetic classification of jumping spiders (Araneae: Salticidae). J. Arachnol..

[20] Xia P.L., Huang Y., Xie X.F., Yan S.Y., Luo Y., Yang W.J. (2021). The complete mitochondrial genome and phylogenetic analysis of *Phintella cavaleriei* (Araneae: Salticidae). Mitochondrial DNA Part B Resour..

[21] Yang D., Yan X., Xu K., Yang W., Li C. (2019). The complete mitochondrial genome of *Epeus alboguttatus* (Araneae: Salticidae). Mitochondrial DNA Part B Resour..

[22] Masta S.E., Boore J.L. (2004). The complete mitochondrial genome sequence of the spider *Habronattus oregonensis* reveals rearranged and extremely truncated tRNAs. Mol. Biol. Evol..

[23] Ye W., Wang J., Zhao X., Liu H., Zhu S. (2022). Mitochondrial genomes of two *Lycosa* spiders (Araneae, Lycosidae): Genome description and phylogenetic implications. Diversity.

[24] Yang W.J., Xu K.K., Yang D.X., Li C. (2019). The complete mitochondrial genome of *Oxyopes hupingensis* (Araneae: Oxyopidae): Characterization and phylogenetic analysis. Mitochondrial DNA Part B Resour..

[25] Wang Z.-L., Huang J., Li M.-Y., Yu X.-P. (2020). The complete mitochondrial genome of a nursery-web spider *Dolomedes angustivirgatus* (Araneae: Pisauridae). Mitochondrial DNA Part B.

[26] Chen C., Xu K., Yan Y., Yang W., Yang H., Li C., Yang D. (2019). The complete mitochondrial genome of a jumping spider, *Cheliceroides longipalpis* Zabka (Araneae: Salticidae). Mitochondrial DNA Part B Resour..

[27] Pan W.J., Fang H.Y., Zhang P., Pan H.C. (2016). The complete mitochondrial genome of pantropical jumping spider *Plexippus paykulli* (Araneae: Salticidae). Mitochondrial DNA.

[28] Kim J.Y., Yoon K.B., Park Y.C. (2016). The complete mitochondrial genome of the jumping spider *Telamonia vlijmi* (Araneae: Salticidae). Mitochondrial DNA.

[29] Fang W.Y., Wang Z.L., Li C., Yang X.Q., Yu X.P. (2016). The complete mitogenome of a jumping spider *Carrhotus xanthogramma* (Araneae: Salticidae) and comparative analysis in four salticid mitogenomes. Genetica.

[30] Pan W.J., Fang H.Y., Zhang P., Pan H.C. (2016). The complete mitochondrial genome of flat spider *Selenops bursarius* (Araneae: Selenopidae). Mitochondrial DNA.

[31] Ding Y., Xu H., Ma H., Zhang J. (2022). The complete mitogenome of *Plator insolens* (Araneae: Trochanteriidae) with phylogenetic implication. Mitochondrial DNA Part B Resour..

[32] Meng G., Li Y., Yang C., Liu S. (2019). MitoZ: A toolkit for animal mitochondrial genome assembly, annotation and visualization. Nucleic Acids Res..

[33] Chen S., Zhou Y., Chen Y., Gu J. (2018). Fastp: An ultra-fast all-in-one FASTQ preprocessor. Bioinformatics.

[34] Nurk S., Meleshko D., Korobeynikov A., Pevzner P.A. (2017). MetaSPAdes: A new versatile metagenomic assembler. Genome Res..

[35] Bernt M., Pütz J., Florentz C., Donath A., Jühling F., Stadler P.F., Externbrink F., Fritzsch G., Middendorf M. (2013). MITOS: Improved de novo metazoan mitochondrial genome annotation. Mol. Phylogenet. Evol..

[36] Laslett D., Canbäck B. (2008). ARWEN: A program to detect tRNA genes in metazoan mitochondrial nucleotide sequences. Bioinformatics.

[37] Zheng S., Poczai P., Hyvönen J., Tang J., Amiryousefi A. (2020). Chloroplot: An online program for the versatile plotting of organelle genomes. Front. Genet..

[38] Zhang D., Gao F., Jakovlić I., Zou H., Zhang J., Li W.X., Wang G.T. (2020). PhyloSuite: An integrated and scalable desktop platform for streamlined molecular sequence data management and evolutionary phylogenetics studies. Mol. Ecol. Resour..

[39] Perna N.T., Kocher T.D. (1995). Patterns of nucleotide composition at fourfold degenerate sites of animal mitochondrial genomes. J. Mol. Evol..

[40] Ranwez V., Douzery E.J.P., Cambon C., Chantret N., Delsuc F. (2018). MACSE v2: Toolkit for the alignment of coding sequences accounting for frameshifts and stop codons. Mol. Biol. Evol..

[41] Zhang Z. (2022). KaKs_Calculator 3.0: Calculating selective pressure on coding and non-coding sequences. Genom. Proteom. Bioinf..

[42] Librado P., Rozas J. (2009). DnaSP v5: A software for comprehensive analysis of DNA polymorphism data. Bioinformatics.

[43] Tamura K., Stecher G., Kumar S. (2021). MEGA11: Molecular evolutionary genetics analysis version 11. Mol. Biol. Evol..

[44] Benson G. (1999). Tandem Repeats Finder: A program to analyze DNA sequences. Nucleic Acids Res..

[45] Nakamura T., Yamada K.D., Tomii K., Katoh K. (2018). Parallelization of MAFFT for large-scale multiple sequence alignments. Bioinformatics.

[46] Capella-Gutiérrez S., Silla-Martínez J.M., Gabaldón T. (2009). TrimAl: A tool for automated alignment trimming in large-scale phylogenetic analyses. Bioinformatics.

[47] Kalyaanamoorthy S., Minh B.Q., Wong T.K.F., Von Haeseler A., Jermiin L.S. (2017). ModelFinder: Fast model selection for accurate phylogenetic estimates. Nat. Methods.

[48] Minh B.Q., Schmidt H.A., Chernomor O., Schrempf D., Woodhams M.D., Von Haeseler A., Lanfear R., Teeling E. (2020). IQ-TREE 2: New models and efficient methods for phylogenetic inference in the genomic era. Mol. Biol. Evol..

[49] Hoang D.T., Chernomor O., Von Haeseler A., Minh B.Q., Vinh L.S. (2018). UFBoot2: Improving the ultrafast bootstrap approximation. Mol. Biol. Evol..

[50] Ronquist F., Teslenko M., Van Der Mark P., Ayres D.L., Darling A., Höhna S., Larget B., Liu L., Suchard M.A., Huelsenbeck J.P. (2012). MrBayes 3.2: Efficient Bayesian phylogenetic inference and model choice across a large model space. Syst. Biol..

[51] Rambaut A., Drummond A.J., Xie D., Baele G., Suchard M.A. (2018). Posterior summarization in Bayesian phylogenetics using Tracer 1.7. Syst. Biol..

[52] Hassanin A., Léger N., Deutsch J. (2005). Evidence for multiple reversals of asymmetric mutational constraints during the evolution of the mitochondrial genome of Metazoa, and consequences for phylogenetic inferences. Syst. Biol..

[53] Wei S.J., Shi M., Chen X.X., Sharkey M.J., van Achterberg C., Ye G.Y., He J.H. (2010). New views on strand asymmetry in insect mitochondrial genomes. PLoS ONE.

[54] Yan X., Xu K.K., Yang D.X., Li C., Yang W.J. (2019). Characterization of the complete mitochondrial genome and phylogenetic analysis of *Leucauge celebesiana* (Araneae: Tetragnathidae). Mitochondrial DNA Part B Resour..

[55] Yang W.J., Xu K.K., Liu Y., Yang D.X., Li C. (2019). Complete mitochondrial genome and phylogenetic analysis of *Argiope perforata* (Araneae: Araneidae). Mitochondrial DNA Part B Resour..

[56] Yan Y., Xu K.K., Yang D.X., Li C., Yang W.J. (2019). The complete mitochondrial genome of *Argiope ocula* (Araneae: Araneidae) and its phylogeny. Mitochondrial DNA Part B Resour..

[57] Kumar V., Tyagi K., Chakraborty R., Prasad P., Kundu S., Tyagi I., Chandra K. (2020). The complete mitochondrial genome of endemic giant tarantula, *Lyrognathus crotalus* (Araneae: Theraphosidae) and comparative analysis. Sci. Rep..

[58] Qiu Y., Song D., Zhou K., Sun H. (2005). The mitochondrial sequences of *Heptathela hangzhouensis* and *Ornithoctonus huwena* reveal unique gene arrangements and atypical tRNAs. J. Mol. Evol..

[59] Kumazawa Y., Miura S., Yamada C., Hashiguchi Y. (2014). Gene rearrangements in gekkonid mitochondrial genomes with shuffling, loss, and reassignment of tRNA genes. BMC Genomics.

[60] Shi W., Miao X.G., Kong X.Y. (2014). A novel model of double replications and random loss accounts for rearrangements in the mitogenome of *Samariscus latus* (Teleostei: Pleuronectiformes). BMC Genomics.

[61] Lorenz C., Lünse C.E., Mörl M. (2017). tRNA modifications: Impact on structure and thermal adaptation. Biomolecules.

[62] Gissi C., Iannelli F., Pesole G. (2008). Evolution of the mitochondrial genome of Metazoa as exemplified by comparison of congeneric species. Heredity (Edinb)..

[63] Dávila S., Piñero D., Bustos P., Cevallos M.A., Dávila G. (2005). The mitochondrial genome sequence of the scorpion *Centruroides limpidus* (Karsch 1879) (Chelicerata; Arachnida). Gene.

[64] Xue X.F., Deng W., Qu S.X., Hong X.Y., Shao R. (2018). The mitochondrial genomes of sarcoptiform mites: Are any transfer RNA genes really lost?. BMC Genomics.

[65] Giegé R., Jühling F., Pütz J., Stadler P., Sauter C., Florentz C. (2012). Structure of transfer RNAs: Similarity and variability. Wiley Interdiscip. Rev. RNA.

[66] Fernández-Silva P., Enriquez J.A., Montoya J. (2003). Replication and transcription of mammalian mitochondrial DNA. Exp. Physiol..

[67] Zhang P., Fang H.Y., Pan W.J., Pan H.C. (2016). The complete mitochondrial genome of the writing spider *Argiope amoena* (Araneae: Araneidae). Mitochondrial DNA.

[68] Zhang P., Fang H.Y., Pan W.J., Pan H.C. (2016). The complete mitochondrial genome of the wasp spider *Argiope bruennichi* (Araneae: Araneidae). Mitochondrial DNA.

[69] Pan W.J., Fang H.Y., Zhang P., Pan H.C. (2016). The complete mitochondrial genome of *Nephila clavata* (Araneae: Nephilidae) Chinese population. Mitochondrial DNA.

[70] Hu M., Jex A.R., Campbell B.E., Gasser R.B. (2007). Long PCR amplification of the entire mitochondrial genome from individual helminths for direct sequencing. Nat. Protoc..

[71] Liu M., Zhang Z., Peng Z. (2015). The mitochondrial genome of the water spider *Argyroneta aquatica* (Araneae: Cybaeidae). Zool. Scr..

[72] Sun J.T., Jin P.Y., Hoffmann A.A., Duan X.Z., Dai J., Hu G., Xue X.F., Hong X.Y. (2018). Evolutionary divergence of mitochondrial genomes in two *Tetranychus* species distributed across different climates. Insect Mol. Biol..

[73] Chang H., Qiu Z., Yuan H., Wang X., Li X., Sun H., Guo X., Lu Y., Feng X., Majid M. (2020). Evolutionary rates of and selective constraints on the mitochondrial genomes of Orthoptera insects with different wing types. Mol. Phylogenet. Evol..

[74] Nielsen R. (2005). Molecular signatures of natural selection. Annu. Rev. Genet..

[75] Shen Y.Y., Liang L., Zhu Z.H., Zhou W.P., Irwin D.M., Zhang Y.P. (2010). Adaptive evolution of energy metabolism genes and the origin of flight in bats. Proc. Natl. Acad. Sci. USA.

[76] Wang H., Meng T., Wei W. (2018). Analysis of synonymous codon usage bias in helicase gene from *Autographa californica* multiple nucleopolyhedrovirus. Genes Genom..

[77] Sueoka N. (1988). Directional mutation pressure and neutral molecular evolution. Proc. Natl. Acad. Sci. USA.

[78] Wright F. (1990). The ‘effective number of codons’ used in a gene. Gene.

[79] Cai Y., Yang X. (2022). Codon usage bias and its influencing factors in the chloroplast genome of *Macadamia integrifolia* Maiden & Betche. Plant Sci. J..

[80] Fernández R., Kallal R.J., Dimitrov D., Ballesteros J.A., Arnedo M.A., Giribet G., Hormiga G. (2018). Phylogenomics, diversification dynamics, and comparative transcriptomics across the spider tree of life. Curr. Biol..

[81] Garrison N.L., Rodriguez J., Agnarsson I., Coddington J.A., Griswold C.E., Hamilton C.A., Hedin M., Kocot K.M., Ledford J.M., Bond J.E. (2016). Spider phylogenomics: Untangling the spider tree of life. PeerJ.

[82] Kallal R.J., Kulkarni S.S., Dimitrov D., Benavides L.R., Arnedo M.A., Giribet G., Hormiga G. (2021). Converging on the orb: Denser taxon sampling elucidates spider phylogeny and new analytical methods support repeated evolution of the orb web. Cladistics.

[83] Azevedo G.H.F., Bougie T., Carboni M., Hedin M., Ramírez M.J. (2022). Combining genomic, phenotypic and Sanger sequencing data to elucidate the phylogeny of the two-clawed spiders (Dionycha). Mol. Phylogenet. Evol..

[84] Maddison W.P., Evans S.C., Hamilton C.A., Bond J.E., Lemmon A.R., Lemmon E.M. (2017). A genome-wide phylogeny of jumping spiders (Araneae, Salticidae), using anchored hybrid enrichment. ZooKeys.

[85] Żabka M. (1985). Systematic and zoogeographic study on the family Salticidae (Araneae) from Viet-Nam. Annales Zoologici, Warszawa.

[86] Logunov D.V. (2021). Jumping spiders (Araneae: Salticidae) of the Na Hang Nature Reserve, Tuyen Quang Province, Vietnam. Arachnology.

