# Supplementary material for: Comparative Mitogenomics of Jumping Spiders with First Complete Mitochondrial Genomes of Euophryini (Araneae: Salticidae)
Source: Insects. 2023 Jun 2;14(6):517. doi: 10.3390/insects14060517 (PMC10299072; doi:10.3390/insects14060517)
Supplement: Supplementary file 1 [file insects-14-00517-s001.zip › SupplementaryMaterial.mod/Fig.S1.pdf]

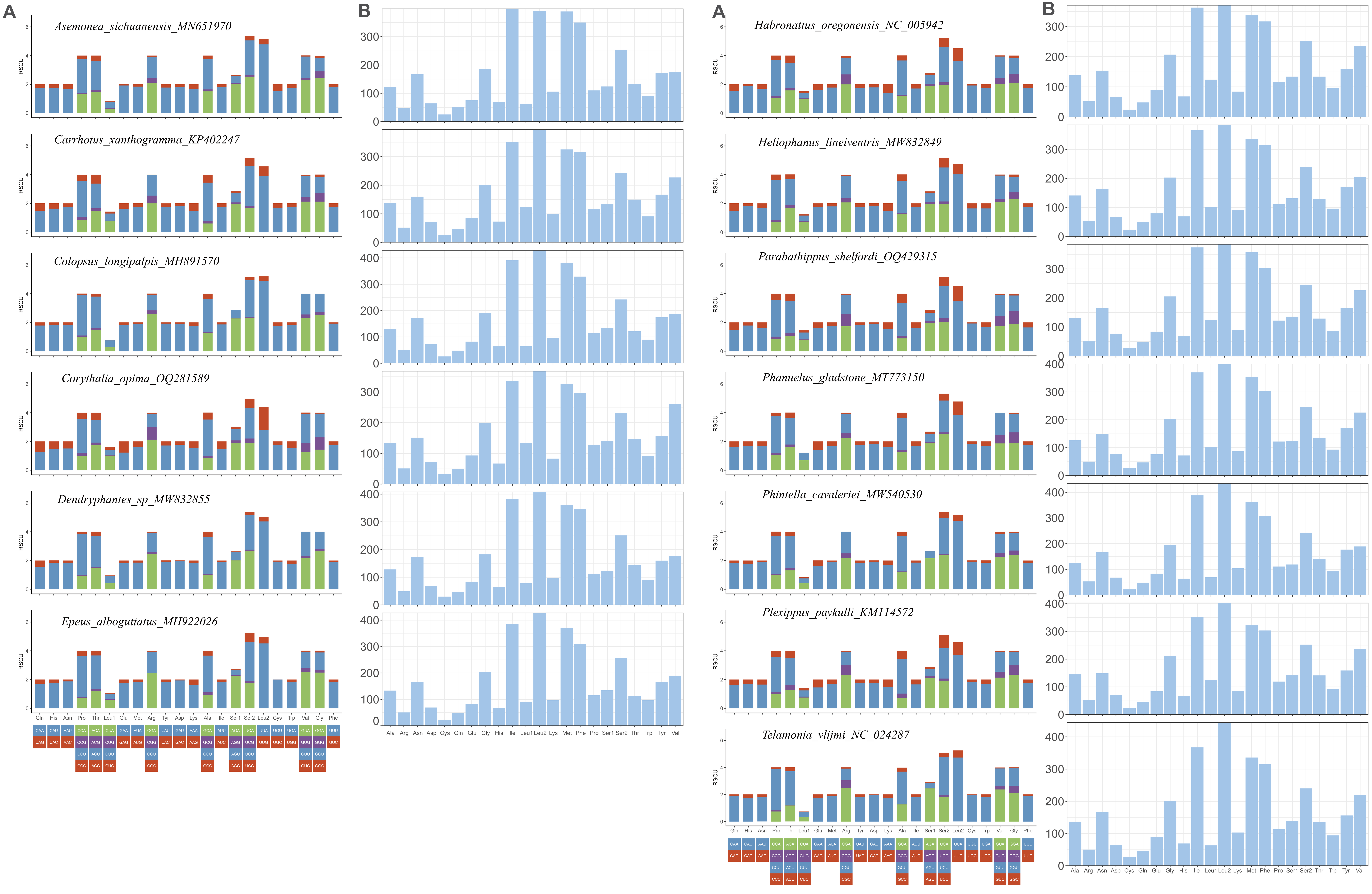

Fig. S1. The (A) relative synonymous codon usage (RSCU) and (B) number of amino acids in the 13 protein coding genes of salticid species. Codon families are labeled below the X-axis.
