# Supplementary material for: Comparative Mitogenomics of Jumping Spiders with First Complete Mitochondrial Genomes of Euophryini (Araneae: Salticidae)
Source: Insects. 2023 Jun 2;14(6):517. doi: 10.3390/insects14060517 (PMC10299072; doi:10.3390/insects14060517)
Supplement: Supplementary file 1 [file insects-14-00517-s001.zip › SupplementaryMaterial.mod/Fig.S2.pdf]

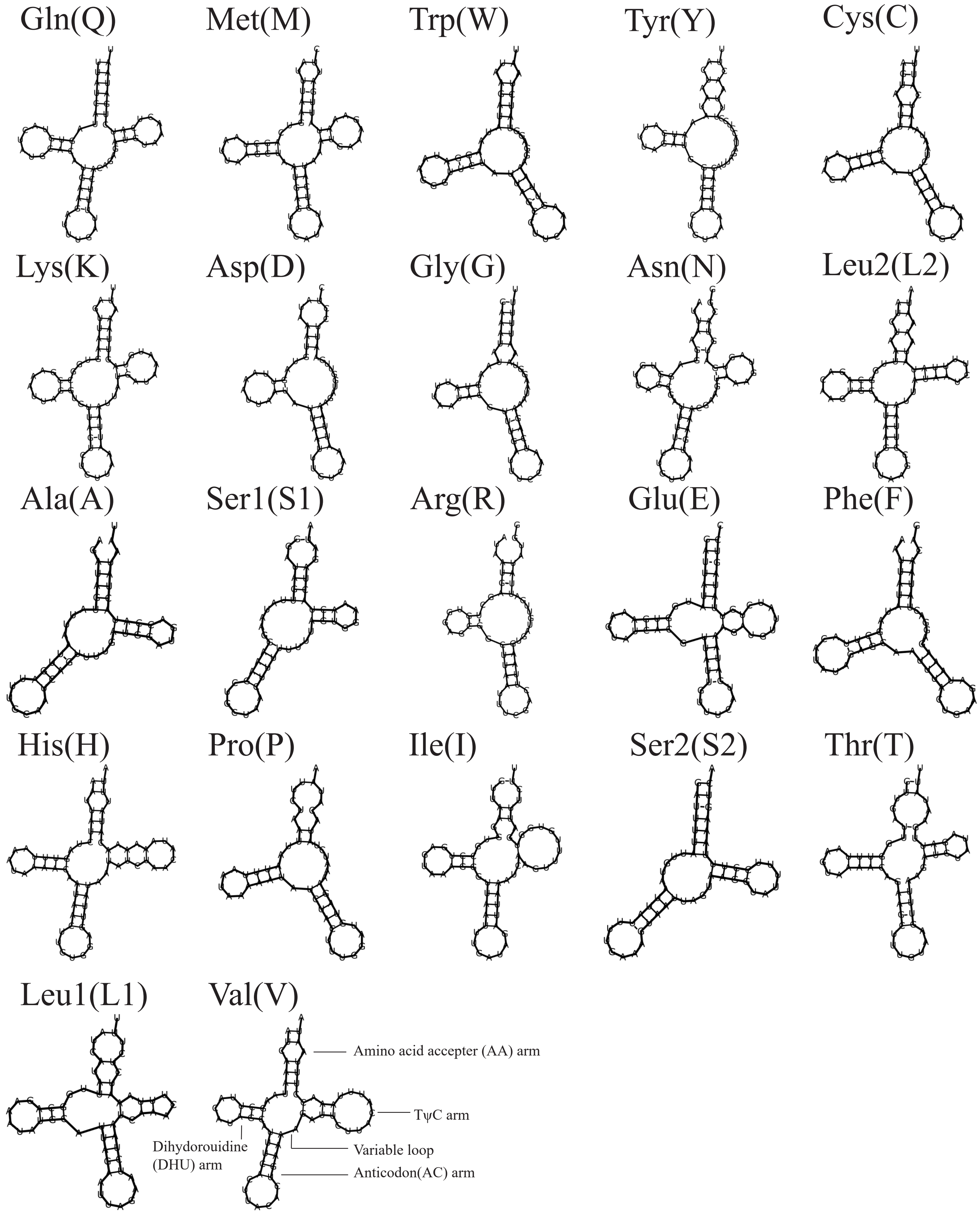
Gln(Q)
Met(M)
Trp(W)
Tyr(Y)
Cys(C)
Lys(K)
Asp(D)
Gly(G)
Asn(N)
Leu2(L2)
Ala(A)
Ser1(S1)
Arg(R)
Glu(E)
Phe(F)
His(H)
Pro(P)
Ile(I)
Ser2(S2)
Thr(T)
Leu1(L1)
Val(V)
Amino acid accepter (AA) arm
TψC arm
Dihydorouidine
(DHU) arm
Variable loop
Anticodon(AC) arm
