# Supplementary material for: Comparative Mitogenomics of Jumping Spiders with First Complete Mitochondrial Genomes of Euophryini (Araneae: Salticidae)
Source: Insects. 2023 Jun 2;14(6):517. doi: 10.3390/insects14060517 (PMC10299072; doi:10.3390/insects14060517)
Supplement: Supplementary file 1 [file insects-14-00517-s001.zip › SupplementaryMaterial.mod/Fig.S4.pdf]

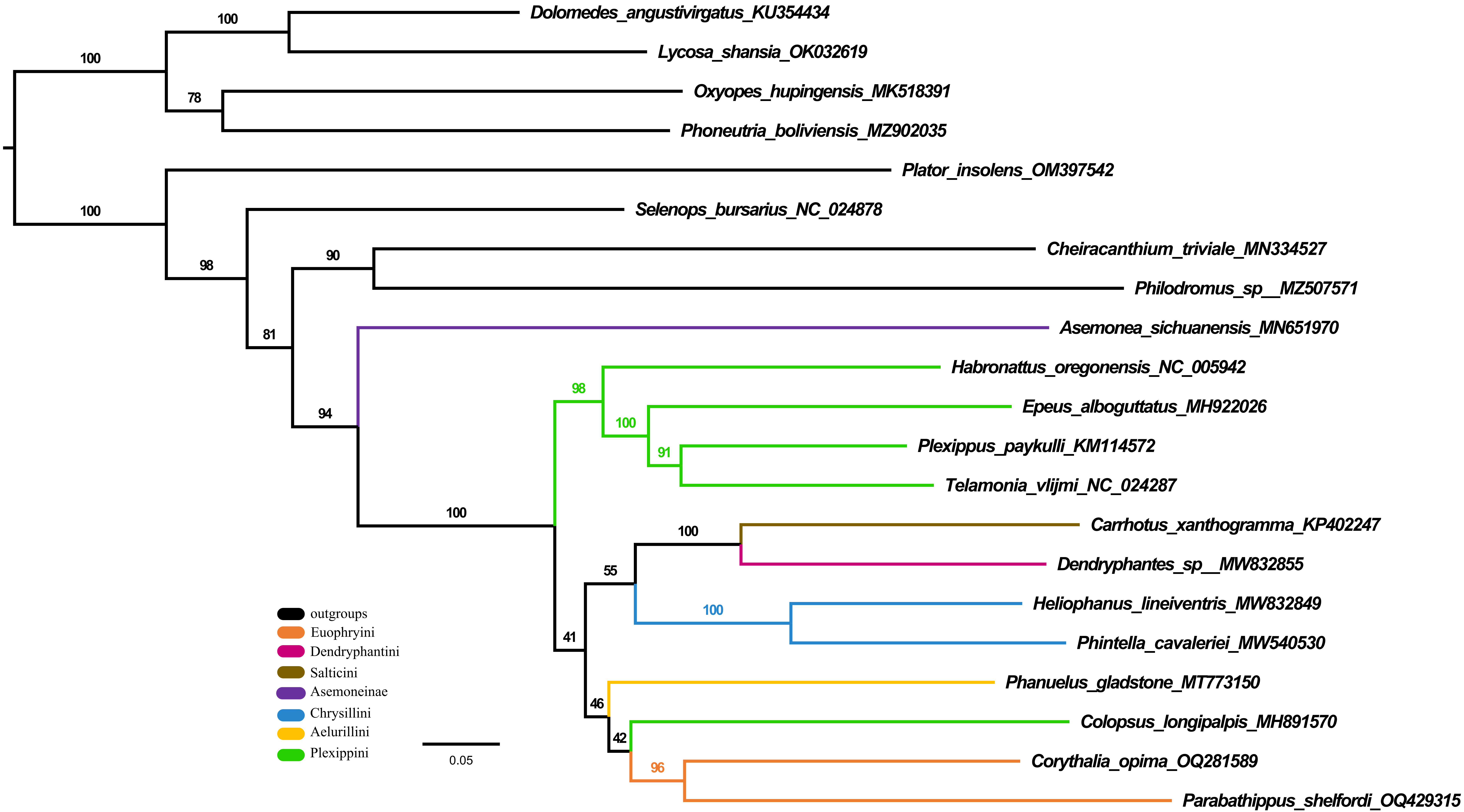


Fig. S4. Phylogenetic tree from the ML analysis on the amino acid dataset, with the numbers on the branches indicate bootstrap supports.
