# Supplementary material for: Comparative Mitogenomics of Jumping Spiders with First Complete Mitochondrial Genomes of Euophryini (Araneae: Salticidae)
Source: Insects. 2023 Jun 2;14(6):517. doi: 10.3390/insects14060517 (PMC10299072; doi:10.3390/insects14060517)
Supplement: Supplementary file 1 [file Fig.S5.pdf › SupplementaryMaterial.mod/Fig.S5.pdf]

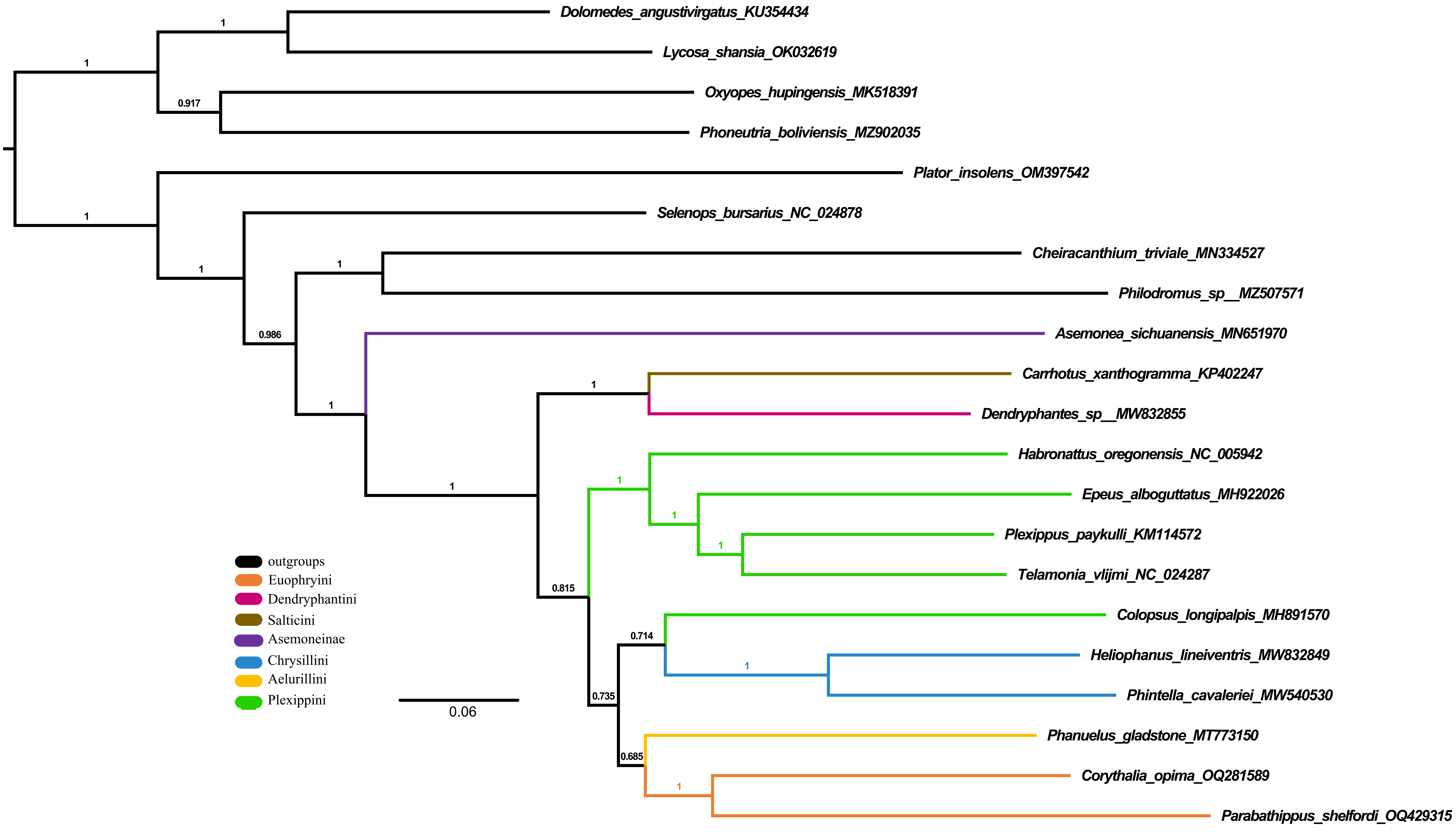

Fig. S5. Phylogenetic tree from the BI analysis on the amino acid dataset, with the numbers on the branches indicate posterior probabilities.
